# 

**Published:** 1899-09-16

**Authors:** 


### NOTICE TO CORRESPONDENTS.

All MS., Letters, Books for Review, and other matters intended for the Editor should be addressed The Editor, "The Hospital," The Hospital Building, 28 & 29, Southampton Street, Strand, W.C.

The Editor cannot undertake to return rejected MS., even when accompanied by stamped directed envelope.

**Notice to Advertisers and Subscribers.**

All Advertisements, Orders for Copies of The Hospital, and Business Communications should be addressed to **THE MANAGER (Not to the Editor)**, The Hospital, The Hospital Building, 28 & 29, Southampton Street, Strand, London, W.C.

The Cost of Subscription, post free, is as follows:—Per week, 2½d.; per quarter, 2s. 8d.; per half-year, 5s. 3d.; per annum, 10s. 6d. Foreign:—Per annum, 15s. 2d., payable, in all cases, in advance.

**NOTICE.—Vol. XXV. of "The Hospital" (October, 1898, to March, 1899), in handsome cloth binding, is now on sale at the Office of this Journal, price 7s. 6d. Cases for binding the Numbers contained in this Volume 1s. 6d. Index to Vol. XXV., 2½d., post free.**

**The Monthly Part for September is now ready. Price 6d., by post 8d.**

### BOOKS RECEIVED.

Henry J. Glaisher.
"Constipation and its Modern Treatment." By George Herschell, M.D. Second edition.

Thomas O. Todd (Sunderland).

"Hints to Probationer Midwives." By Annie S. Pruett.

Robert Anderson (Glasgow).
"Eighth Annual Report." By John Wilson, M.D., D.P.H., Medical Officer to the County of Lanark.

West, Newman, and Co.
"Archives of Surgery." By Jonathan Hutchinson, LL.D., F.R.S.

Cassell and Co.

"Orthopædic Surgery." By J. Jackson Clarke, M.B., F.R.C.S.

George Barber.
"A Handbook with Hints for the Nursery." By J. Maclean Carvell, M.R.C.S.

J. W. Wakeham.
"Annual Report." By N. C. Collier, Medical Officer of Health for the Parish of Hammersmith.

### SCRAPS AND GLEANINGS.

During the past thirty years 78,966 persons have been admitted to the Dunoon Seaside Homes.

During last year upwards of 2,700 visits were made to the homes of out-patients at the Lewes Infirmary.

Since the foundation of the Hereford Infirmary 50,000 in-patients have been treated, and 121,000 out-patients.

The total of this year's Hospital Saturday and Sunday Fund at Bournemouth was nearly £20 in excess of last year's.

The Stockton and Thornaby Hospital have in hand and promised a sum of £2,750 towards the enlargement of the hospital.

Nine hundred and fifty-eight patients have been admitted to the Metropolitan Asylums Board Hospitals during the last fortnight.

The discussion and decision as to the proposed improvement and extension of the Leeds Workhouse Infirmary has been deferred till October.

The donations to the Alexandra Hospital, Woodhall Spa, during the past year amounted to £229 19s., of which £200 has been placed to the building fund.

Swindon Hospital Saturday has this year broken its record, the amount handed over to the Swindon Victoria Hospital, after deducting all expenses, being £138 8s. 7d.

The number of patients admitted to the Surrey Convalescent Home, Seaford, during the past year, *i.e.*, 662, is the largest number admitted in any one year since the home was opened. The year's working, however, shows a deficit of £265 8s., owing to a falling off in the receipts as compared with 1897-1898, the expenditure for that year having been greater than in the year 1898-99.

The new consumptive sanatorium to be erected on Middle Deeside on the lines of Dr. Otto Walther's institution in Germany is to provide accommodation for at least forty patients. The promoters of the scheme, who are advocates of Aberdeen, have made a statement, in consequence of inaccurate paragraphs which have appeared, that the scheme was originally set on foot in response to the wishes of a number of the prominent consulting physicians of Scotland.

The building of the additional nurses' house to the Salisbury Infirmary is now approaching completion, but funds are urgently needed for this purpose. It is now more than 10 years since the work was decided upon by the governors.

Eight hundred and five patients have been admitted during the past year to the Salisbury Infirmary, against 796 in the previous year; and 1,390 out-patients were treated, against 1,472 in 1897-98.

The captain of the barque "Windrush," of Sutherland, whilst at Ostend with his vessel collected £22 from visitors to his barque, which he gave in equal proportions to an Ostend society for collecting money for the poor and to the Sutherland Infirmary.

At a recent meeting of the Dewsbury District Joint Hospital Board the estimates for the erection of the joint hospital, amounting to upwards of £26,000, were considered, but as this amount is over £4,000 in excess of the borrowing powers sanctioned by the Local Government Board the report was referred back to the sub-committee to see if they could curtail the cost.

In consequence of the outbreak of diphtheria at Woking, and the insufficient accommodation for its treatment already in existence, it has been decided to erect an iron building of two wards, each ward to contain 12 patients, at a cost of about £475, including the foundation walls, such building to be completed within 14 days from the date of the possession of the foundations.

The deficiency during the past year on the income side of the accounts of the Sheffield Royal Infirmary is the largest since 1894, *i.e.*, £2,706 odd, and an urgent appeal was made by the Lord Mayor of Sheffield at the annual meeting of the infirmary on the 6th. During the past year there were 2,917 in-patients, 5,072 out-patients, 14,244 casualties, and 1,986 dental cases, being an increase of 676 in-patients over the preceding year.

The intended buildings of the proposed small-pox hospital for Leeds, for the erection of which the City Council have applied to the Local Government Board to sanction a loan of £21,250, are to contain accommodation for 104 patients. In addition to the main structure there are to be isolation pavilions. The designer of the plans is Mr. E. T. Hall, of Moorgate Street, who estimates the total cost of the works at £61,781.

### THE STUDENT OF MEDICINE.

### APPOINTMENTS.

T. S. Balfour, M.D.Edin., M.B., C.M., appointed Medical Officer and Public Vaccinator to the North Chardstock District of the Axminster Union. C. S. Brebner, M.B., Ch.B.Edin., appointed House Surgeon to the Durham County Hospital. George H. Edington, M.D., M.R.C.S.Eng., L.R.C.P.Lond., F.F.P.S.Glasg., appointed Extra Dispensary Surgeon, Royal Hospital for Sick Children, Glasgow. C. R. Kendal, M.R.C.S.-Eng., and L.S.A., appointed Medical Officer for the Parish of Wendron, in the Helston Union. T. Noy Leah, M.R.C.S., L.R.C.P.Lond., appointed Surgeon to the Provident Dispensary of the Royal Albert Hospital. T. H. Livingstone, M.B., Ch.B.-Edin., appointed House Surgeon to the Rochdale Infirmary. J. J. Nixon Morris, M.R.C.S., L.R.C.P.Lond., appointed Assistant Surgeon to the Royal Albert Hospital, Devonport. D. G. Macleod Munro, M.B., M.R.C.P.Edin., appointed Physician to the Cheltenham Provident Dispensary. E. Robinson, M.D.Edin., M.B., C.M., appointed Medical Officer of the Frencham District of the Farnham Union. T. W. Tetley, M.R.C.S., L.R.C.P., appointed House Physician to the General Infirmary, Leeds. H. L. Thurnell, M.A.Camb., B.A., M.R.C.S., L.R.C.P.Lond., appointed Medical Officer of the Gravesend District of the Gravesend and Milton Union.

St. Thomas's Hospital.—The following gentlemen have been appointed House Officers from September 5th. House Physicians: E. H. Ross, L.R.C.P., M.R.C.S.; J. Gaff, L.R.C.P., M.R.C.S.; A. Bevan, L.R.C.P., M.R.C.S.; and H. C. Thorp, M.A., M.B., B.C.Cantab. Assistant House Physicians: F. H. Ellis, B.A., M.B., B.C.Cantab., L.R.C.P., M.R.C.S.; and B. F. Howlett, L.R.C.P., M.R.C.S. House Surgeons: H. J. Phillips, L.R.C.P., M.R.C.S.; P. W. G. Sargent, M.A., M.B., B.C.Cantab., L.R.C.P., M.R.C.S.; S. A. Lucas, L.R.C.P., M.R.C.S.; and H. T. D. Acland, L.R.C.P., M.R.C.S. Assistant House Surgeons: A. Webb Jones, L.R.C.P., M.R.C.S.; E. A. Gates, L.R.C.P., M.R.C.S.; E. C. Bourdas, L.R.C.P., M.R.C.S.; and N. Unsworth, L.R.C.P., M.R.C.S. Obstetric House Physicians: (Senior) H. M. Scraping, B.A.Cantab., L.R.C.P., M.R.C.S.; and (Junior) A. E. Stevens, M.B.Durh., L.R.C.P., M.R.C.S. Ophthalmic House Surgeons: (Senior) T. Hoban, L.R.C.P., M.R.C.S.; and (Junior) J. A. Barnes, L.R.C.P., M.R.C.S. Clinical Assistants in the Special Department for Diseases of the Throat: L. H. Lindley, B.A., M.B., B.Ch.Oxon., and C. L. Hawkins, B.A.-Cantab., L.R.C.P., M.R.C.S. Skin: H. R. Beale, L.R.C.P., M.R.C.S., and T. Perrin, L.R.C.P., M.R.C.S. Ear: A. E. Martin, B.A.Cantab., L.R.C.P., M.R.C.S.

